# Harnessing Nanotechnology: Emerging Strategies for Multiple Myeloma Therapy

**DOI:** 10.3390/biom14010083

**Published:** 2024-01-09

**Authors:** Min Yang, Yu Chen, Li Zhu, Liangshun You, Hongyan Tong, Haitao Meng, Jianpeng Sheng, Jie Jin

**Affiliations:** 1 Department of Hematology, The First Affiliated Hospital, Zhejiang University School of Medicine, Hangzhou 310003, China; yangmin111111@zuaa.zju.edu.cn (M.Y.); chenyu@zuaa.zju.edu.cn (Y.C.); julia28@zju.edu.cn (L.Z.); youliangshun@zju.edu.cn (L.Y.); tonghongyan@zju.edu.cn (H.T.); menghait2004@zju.edu.cn (H.M.); 2 Zhejiang Provincial Key Laboratory of Hematopoietic Malignancy, Zhejiang University, Hangzhou 310027, China; 3 Zhejiang Provincial Clinical Research Center for Hematological Disorders, Hangzhou 310003, China; 4 Zhejiang University Cancer Center, Hangzhou 310029, China; shengjp@zju.edu.cn

**Keywords:** multiple myeloma, nanotechnology, nanoparticle-based drug delivery, nano-immunotherapy

## Abstract

Advances in nanotechnology have provided novel avenues for the diagnosis and treatment of multiple myeloma (MM), a hematological malignancy characterized by the clonal proliferation of plasma cells in the bone marrow. This review elucidates the potential of nanotechnology to revolutionize myeloma therapy, focusing on nanoparticle-based drug delivery systems, nanoscale imaging techniques, and nano-immunotherapy. Nanoparticle-based drug delivery systems offer enhanced drug targeting, reduced systemic toxicity, and improved therapeutic efficacy. We discuss the latest developments in nanocarriers, such as liposomes, polymeric nanoparticles, and inorganic nanoparticles, used for the delivery of chemotherapeutic agents, siRNA, and miRNA in MM treatment. We delve into nanoscale imaging techniques which provide spatial multi-omic data, offering a holistic view of the tumor microenvironment. This spatial resolution can help decipher the complex interplay between cancer cells and their surrounding environment, facilitating the development of highly targeted therapies. Lastly, we explore the burgeoning field of nano-immunotherapy, which employs nanoparticles to modulate the immune system for myeloma treatment. Specifically, we consider how nanoparticles can be used to deliver tumor antigens to antigen-presenting cells, thus enhancing the body’s immune response against myeloma cells. In conclusion, nanotechnology holds great promise for improving the prognosis and quality of life of MM patients. However, several challenges remain, including the need for further preclinical and clinical trials to assess the safety and efficacy of these emerging strategies. Future research should also focus on developing personalized nanomedicine approaches, which could tailor treatments to individual patients based on their genetic and molecular profiles.

## 1. Introduction

Multiple myeloma, commonly known as myeloma, is a hematological malignancy characterized by the clonal proliferation of malignant plasma cells in the bone marrow and the production of monoclonal immunoglobulins, or M proteins, that can be detected in the blood or urine [1]. This cancer represents approximately 1% of all cancers and about 10% of all hematologic malignancies [2].

Globally, the incidence of myeloma is approximately 2.1 per 100,000 people annually, with a lifetime risk of 0.7% [3]. Prevalence rates vary across different regions and demographics, with higher rates observed in Western countries, particularly among the elderly and African-American populations [3]. For instance, in the United States, the incidence rate is higher at approximately 6.5 per 100,000 people annually [4]. It is imperative to note that the incidence and mortality rates have been increasing over the past decade, underscoring the urgent need for improved treatment strategies [5].

Multiple myeloma arises from the malignant transformation and unchecked proliferation of plasma cells within the bone marrow. Under normal conditions, plasma cells are responsible for producing antibodies, playing a crucial role in the immune response [6]. However, in multiple myeloma, these cells turn cancerous and multiply excessively, disrupting the bone marrow’s healthy function [7]. The pathogenesis of multiple myeloma is characterized by the malignant transformation of B-cell progenitors in the bone marrow microenvironment. These myeloma cells express a variety of cell surface receptors and proteins that can be exploited for targeted therapies. Notable among these are CD38, CD138, and the B-cell maturation antigen (BCMA), which have been successfully targeted by monoclonal antibodies in the treatment of multiple myeloma [8].

During normal hematopoiesis, B-cell progenitors undergo a series of well-orchestrated maturation events, transitioning through various stages including the pro-B cell, pre-B cell, immature B cell, and mature B cell stages before finally differentiating into antibody-secreting plasma cells. This maturation is accompanied by critical genetic rearrangements and somatic hypermutation, both of which are essential for generating a diverse antibody repertoire [9].

In the case of multiple myeloma, the transformation typically occurs at the later stages of B-cell development. The exact point of malignant transformation can vary, but it is generally believed to occur during the transition from a mature B-cell to an early plasma cell. This stage is marked by further genetic alterations, including translocations involving the immunoglobulin heavy chain locus or oncogenic mutations that confer a proliferative advantage to the cells. The resultant clonal plasma cells then accumulate in the bone marrow, leading to the characteristic features of multiple myeloma such as osteolytic lesions, anemia, and renal dysfunction [10].

These malignant plasma cells retain some functional characteristics of normal plasma cells, such as antibody secretion; however, they produce a monoclonal protein that can be detected in the serum or urine of patients and is used as a biomarker for the disease [11].

Myeloma cells produce excessive amounts of a single type of antibody known as monoclonal protein (M protein), which is not effective in fighting infections and can lead to complications such as kidney damage [12]. Furthermore, the proliferation of myeloma cells in the bone marrow creates an imbalance, interfering with the production of normal blood cells. This imbalance can lead to anemia (a shortage of red blood cells), thrombocytopenia (a shortage of platelets), and leukopenia (a shortage of white blood cells), each of which can contribute to the symptoms and complications associated with the disease [13].

Moreover, myeloma cells alter the bone marrow microenvironment to support their survival and growth, leading to bone lesions and increased production of osteoclasts, cells that break down bone tissue. These processes contribute to the bone pain and fractures often seen in myeloma patients [14]. In a concise yet impactful study, researchers have elucidated the role of multiple myeloma (MM)-derived exosomes in exacerbating bone disease by promoting osteoclast (OC) differentiation, a hallmark of MM pathology. The study revealed that these exosomes facilitate the transformation of preosteoclasts into bone-resorbing cells, with implications observed across murine models and human OCs, as well as corroborated by patient sera. While those findings significantly advance our understanding of MM-induced skeletal complications, they beckon further investigation to decode the molecular cargo of these exosomes, potentially offering novel therapeutic targets for MM-associated bone disease [15].

Another study investigates osteocyte involvement in the pathogenesis of bone lesions and osteoclast (OCL) activation in multiple myeloma (MM). Findings indicate a significant reduction in viable osteocytes in MM patients, particularly those with bone lesions, where increased apoptosis is evident. In vitro experiments show that MM cells induce preosteocyte apoptosis and alter their gene expression to favor osteoclastogenic activity, notably through upregulation of interleukin (IL)-11. These results highlight a potential mechanistic link between reduced osteocyte viability and MM-related bone complications, suggesting a new avenue for therapeutic exploration [16].

**Figure 1:**
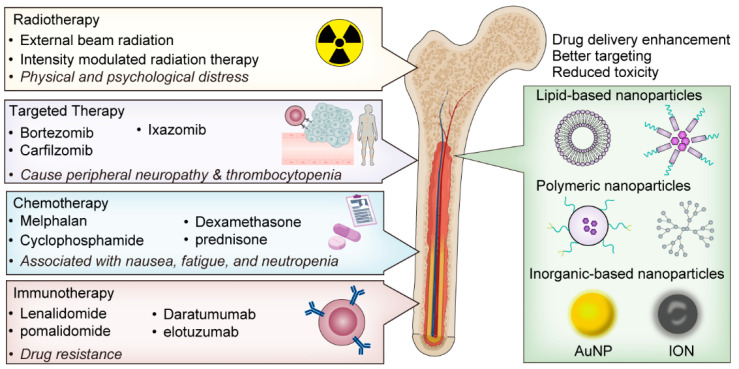
Current treatment options for myeloma and potential of nanotechnology.

The current standard of care for multiple myeloma is complex and typically involves a multimodal approach. First-line treatment usually includes a combination of chemotherapy, corticosteroids, and targeted therapies. Chemotherapy drugs, such as melphalan and cyclophosphamide, are used to kill rapidly dividing cells, while corticosteroids like dexamethasone and prednisone help reduce inflammation and affect the immune system (Figure 1).

Targeted therapies have revolutionized myeloma treatment in recent years. Proteasome inhibitors, such as bortezomib, carfilzomib, and ixazomib, disrupt the proteasome pathway, a crucial cellular machinery for protein degradation that myeloma cells heavily rely on. Immunomodulatory drugs, including lenalidomide and pomalidomide, modulate the immune system and have direct anti-tumor effects [4]. Monoclonal antibodies like daratumumab and elotuzumab target specific proteins on myeloma cells, leading to cell death. Bispecific antibodies, exemplified by agents such as Elranatamab, Teclistamab, and Talquetamab, constitute an innovative class of immunotherapies in the management of multiple myeloma. These therapeutics are engineered to concurrently engage with distinct antigens expressed on myeloma cells—BCMA for Teclistamab and GPRC5D for Talquetamab—and CD3 on T-cells. This dual engagement is crucial for the direct recruitment and activation of cytotoxic T-cells at the tumor site, thereby potentiating the immune system’s capacity to selectively and effectively eradicate myeloma cells [17].

For eligible patients, autologous stem cell transplantation—the transplant of the patient’s own stem cells after high-dose chemotherapy—remains a standard of care, often leading to prolonged remission. Furthermore, immunotherapies, particularly chimeric antigen receptor T-cell (CAR-T) therapies, are emerging as promising options for relapsed or refractory myeloma. Supportive treatments, including bone-modifying drugs and treatments for anemia, are also integral to managing symptoms and complications associated with myeloma.

Despite the significant advances in the treatment of multiple myeloma, there are still several challenges that limit the efficacy of current treatment options. One such challenge is the side effects associated with these treatments. Chemotherapy, while a cornerstone in multiple myeloma treatment, brings a well-documented array of side effects such as nausea, fatigue, and neutropenia [18]. The emergence of targeted therapies has ushered in a shift in adverse effect profiles, with proteasome inhibitors like bortezomib inducing peripheral neuropathy and thrombocytopenia, distinguishing them from traditional chemotherapy-related toxicities [19]. Advanced modalities like chimeric antigen receptor (CAR) T-cell therapy and bispecific antibodies introduce further unique side effects by engaging the immune system against myeloma cells, leading to cytokine release syndrome (CRS), neurotoxicity or ICANS (also known as immune effector cell-associated neurotoxicity syndrome), hematologic complications, and heightened infection risks due to prolonged cytopenias [20]. These effects stem from the distinct active mechanisms of these treatments; CAR T-cell therapy’s potent immune activation through modified T-cells and bispecific antibodies’ T-cell redirection to tumor cells can both result in CRS [21]. The side effect landscape of these therapies is not static but dynamic, influenced by evolving treatment regimens, advancements in supportive care, and individual patient factors.

Another major challenge is the development of drug resistance. Multiple myeloma cells can adapt to the therapeutic pressure and evolve mechanisms to evade these treatments, leading to refractory disease [22]. This is a particular issue with proteasome inhibitors and immunomodulatory drugs, where resistance can develop over time [23].

High relapse rates are also a significant concern. Even with stem cell transplantation, the majority of patients will eventually relapse, highlighting the need for more effective maintenance therapies [24]. Furthermore, the impact of treatments on patient quality of life cannot be underestimated. The chronic nature of multiple myeloma and the long-term use of treatments can lead to physical and psychological distress, impacting patients’ well-being and overall quality of life [25].

In summary, while current treatment options have improved survival rates and disease management, there are still considerable challenges that need to be addressed to further improve patient outcomes in multiple myeloma.

Nanotechnology, a rapidly progressing field at the intersection of materials science and biotechnology, has introduced a new paradigm in cancer therapeutics [26]. It involves the manipulation of materials at the nanoscale, typically between 1 and 100 nanometers, to create novel structures and devices [27]. In medicine, particularly in cancer treatment, nanotechnology holds immense potential to revolutionize our approach to disease management (Figure 1).

One of the key advantages of nanotechnology lies in improving drug delivery. Nanoparticles can be engineered to carry therapeutic agents, such as chemotherapeutic drugs or genes, and deliver them directly to the tumor site [28,29]. This targeted delivery can enhance the drug’s concentration at the tumor while minimizing its distribution to healthy tissues, thereby reducing systemic toxicity and improving treatment efficacy [29,30].

Furthermore, nanotechnology can also enable the controlled release of drugs, allowing for sustained drug exposure and reducing the frequency of administration [29,30]. Additionally, through surface modifications, nanoparticles can be designed to evade the immune system, prolong circulation time, and enhance cellular uptake, thereby significantly improving the effectiveness of treatments [29,31].

In summary, nanotechnology offers a promising approach to overcome some of the current limitations in cancer treatment, providing innovative strategies for drug delivery, minimizing side effects, and enhancing treatment effectiveness.

The potential of nanotechnology to transform the treatment landscape for multiple myeloma is increasingly being recognized [31]. One of the challenges in myeloma therapy, as mentioned earlier, is drug resistance. Nanotechnology can help overcome this hurdle by enabling the co-delivery of multiple drugs within a single nanoparticle, allowing for simultaneous targeting of different pathways and reducing the likelihood of resistance development [32].

For instance, a preclinical study demonstrated that dual-drug loaded nanoparticles could effectively kill myeloma cells while sparing healthy cells, suggesting a promising strategy to enhance treatment efficacy and reduce systemic toxicity [33]. In a phase 1 clinical trial, nanoliposomal doxorubicin demonstrated a favorable safety profile and showed preliminary evidence of efficacy in relapsed or refractory multiple myeloma patients [34].

Nanotechnology could also address the challenge of high relapse rates by enabling the delivery of maintenance therapies in a more controlled and sustained manner, potentially improving long-term disease control [35]. Furthermore, by enhancing targeted delivery and reducing systemic exposure, nanotechnology could potentially improve the quality of life of patients by minimizing the side effects associated with long-term treatments [36].

Thus, nanotechnology presents an exciting avenue for the development of more effective, safer, and patient-friendly therapeutic options for multiple myeloma, with the potential to address some of the current challenges in its treatment.

This review aims to provide a comprehensive overview of the current state and future prospects of nanotechnology in multiple myeloma treatment. In the subsequent sections, we will delve into the recent advancements in nanotechnology that have shown promise in preclinical and clinical myeloma studies [37]. We will discuss how nanoparticles have been used to improve drug delivery, reduce side effects, and enhance the efficacy of myeloma treatments.

Furthermore, we will examine the emerging role of nanotechnology in immunotherapy for myeloma [38]. The use of nanoparticles as carriers for antigens, adjuvants, or immune checkpoint inhibitors has shown potential in enhancing immune responses and overcoming immunosuppression, thus opening up new avenues for myeloma treatment [39].

We will also provide illustrative case studies that highlight the successful application of nanotechnology in myeloma therapy. Additionally, we will discuss the future perspectives, including potential strategies for nanoparticle design and optimization, and the integration of nanotechnology with other cutting-edge technologies such as gene editing and artificial intelligence [40].

Lastly, we will address the potential challenges and roadblocks in the translation of nanotechnology from bench to bedside, such as safety issues, scalability, regulatory hurdles, and the need for a multidisciplinary approach to overcome these challenges [41].

We hope that this review will serve as a comprehensive resource for researchers in the field, providing insights into the potential of nanotechnology to revolutionize myeloma treatment and the steps needed to realize this potential.

## 2. Basics of Nanotechnology in Cancer Treatment

Nanotechnology, the manipulation, creation, and utilization of materials at the nanoscale level [42], represents a revolutionary approach with significant potential in the realm of cancer therapeutics. Nanoparticles, typically sized between 1 and 100 nanometers, possess unique physicochemical properties that are largely dictated by their size, shape, and surface characteristics [43]. These properties include increased surface area to volume ratio, quantum effects, and the ability to carry and deliver a variety of molecules [44].

### 2.1. Nanoparticles for Drug Delivery

In the context of cancer treatment, nanotechnology has emerged as a powerful tool to enhance the delivery of therapeutic agents [45]. Nanoparticles can be designed to carry drugs, genes, and imaging agents, and to release them in a controlled manner at the tumor site [45]. This targeted approach not only improves the selectivity of cancer treatments but also enhances their efficacy by ensuring that the therapeutic agents reach the tumor cells in optimal concentrations [46].

**Figure 2:**
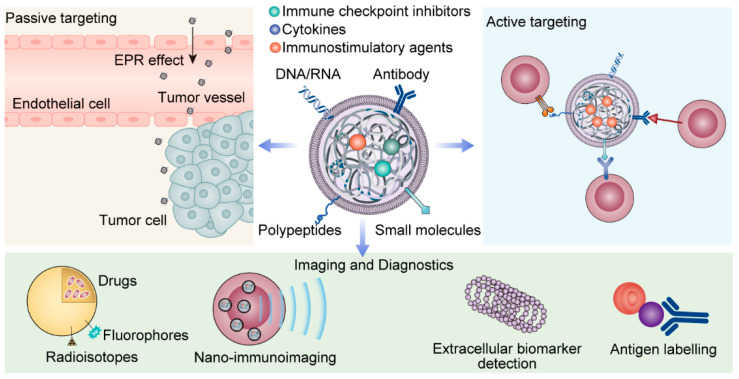
Advantages and application scenarios of nanotechnology.

In essence, nanotechnology holds the potential to revolutionize cancer treatment by addressing some of the limitations of traditional therapies, such as non-specificity, low bioavailability of drugs, and systemic side effects. Through the precise delivery and release of therapeutic agents, nanotechnology could potentially improve the survival and quality of life of cancer patients (Figure 2).

Nanoparticle-based drug delivery systems have emerged as a promising means to enhance the efficacy and tolerability of anticancer drugs [41]. These systems include liposomes, micelles, dendrimers, and nanocrystals, each with unique characteristics and mechanisms of action (Figure 2).

Liposomes are spherical vesicles with an aqueous core enclosed by one or more phospholipid bilayers, which can encapsulate both hydrophilic and lipophilic drugs [47]. Micelles, formed by self-assembly of amphiphilic molecules, are another type of carrier that can increase the solubility and stability of hydrophobic drugs [48].

Dendrimers, highly branched polymers with a high degree of molecular uniformity, can carry drugs either in their interior cavities or on their surface [49]. Nanocrystals, on the other hand, are pure drug particles that are nano-sized, enhancing the dissolution rate and thus the bioavailability of poorly soluble drugs [50].

These nanoparticle-based systems can deliver drugs to tumor cells through two primary mechanisms: passive and active targeting. Passive targeting exploits the enhanced permeability and retention (EPR) effect, a phenomenon where nanoparticles preferentially accumulate in tumor tissue due to its leaky vasculature and poor lymphatic drainage [51].

Active targeting, meanwhile, involves the modification of nanoparticle surfaces with ligands that can bind to specific receptors overexpressed on cancer cells, ensuring a more precise drug delivery [52]. Both strategies aim to increase the concentration of drugs at the tumor site, thereby maximizing therapeutic efficacy and minimizing systemic toxicity. Active targeting strategies in nanoparticle design exploit the unique expression profiles of cell surface markers on myeloma cells to achieve enhanced specificity and therapeutic efficacy. For instance, CD38, a transmembrane glycoprotein highly expressed on myeloma cells, has been effectively targeted by nanoparticles conjugated with anti-CD38 antibodies. Similarly, nanoparticles can be functionalized with ligands for CD138 or BCMA, which are also overexpressed on the surface of myeloma cells. These targeted nanoparticles can then preferentially bind to and be internalized by myeloma cells, ensuring that the cytotoxic agents they carry are delivered directly to the tumor site, thereby minimizing off-target effects and improving patient outcomes [8].

Nanotechnology has been instrumental in advancing targeted therapies for cancer, providing new avenues for the delivery of small molecule inhibitors, monoclonal antibodies, and gene therapies [41].

Small molecule inhibitors, such as tyrosine kinase inhibitors, can be encapsulated into nanoparticles to increase their solubility, stability, and cellular uptake, thereby enhancing their bioavailability [30]. Similarly, monoclonal antibodies, which are proteins that can specifically bind to cancer cell antigens, can be conjugated to nanoparticles to improve their pharmacokinetics and reduce off-target effects [53].

For gene therapies, which involve the delivery of nucleic acids to modify or replace defective genes, nanoparticles can protect the genetic material from degradation and facilitate its transport across physiological barriers [54]. Nanoparticles can also help overcome the challenges of intracellular delivery, which is particularly important for therapies targeting intracellular oncogenic pathways [55]. For example, a novel study presents a promising therapeutic strategy for multiple myeloma (MM), focusing on overcoming drug resistance by targeting the bone marrow microenvironment instead of the cancer cells directly. Researchers developed a nanoparticle-based delivery system capable of carrying small interfering RNA (siRNA) to silence cyclophilin A, a protein implicated in MM progression. Their approach not only hindered MM spread but also, when used in combination with the FDA-approved drug bortezomib, significantly increased survival in a mouse model. This siRNA nanoparticle platform holds potential as a versatile treatment for MM and other malignancies that home to the bone marrow [56]. Another study investigates the use of chitosan/PLGA nanoparticles as carriers for miR-34a, aiming to enhance the biopharmaceutical properties of this microRNA in cancer therapy. These nanoplexes, with a mean diameter of about 160 nm and a positive surface charge, demonstrated high entrapment efficiency and resistance to RNase degradation. They showed potent in vitro anti-tumor effects on multiple myeloma cells and improved transfection efficiency. In vivo, miR-34a-loaded nanoparticles markedly reduced tumor growth in human multiple myeloma xenografts in NOD-SCID mice, leading to increased survival, with high levels of the miRNA detected in the tumors. The nanoplexes remained stable with no toxicity observed, underscoring their potential as a safe and effective delivery system for microRNA-based cancer therapies [57]. Moreover, one study explores a therapeutic approach for multiple myeloma (MM), a hematological malignancy with poor prognosis due to its symbiotic relationship with the bone marrow microenvironment. Interactions involving adhesion receptors and homing factors, such as E-selectin (ES) and cyclophilin A (CyPA), are found to support MM cell homing, proliferation, and drug resistance. The study posits that RNA interference (RNAi) silencing of ES and CyPA could disrupt these interactions, offering a strategy to inhibit MM cell colonization and spread. While small molecule inhibitors and blocking antibodies have proven ineffective or counterproductive, targeting ES and CyPA with RNAi presents a promising avenue for impeding MM progression [58].

Furthermore, by leveraging the enhanced permeability and retention (EPR) effect and active targeting strategies, nanoparticles can selectively deliver these therapeutic agents to tumor cells, minimizing damage to healthy tissues and reducing systemic side effects [51]. In essence, nanotechnology not only enhances the delivery of targeted therapies but also maximizes their therapeutic potential, paving the way for more effective and tolerable cancer treatments.

Nanotechnology-based treatments offer several advantages over traditional methods, providing improved pharmacokinetics and pharmacodynamics, the potential for combination therapies, reduced toxicity, and enhanced patient compliance [30].

Nanoparticles can improve the pharmacokinetics of drugs by increasing their stability, bioavailability, and half-life in the body [59]. For instance, Doxil, the PEGylated liposomal formulation of doxorubicin, exhibits a significantly extended half-life compared to free doxorubicin. While conventional doxorubicin has a plasma half-life of approximately 5 to 10 min due to rapid clearance, Doxil extends the half-life to approximately 55 h. This remarkable increase is primarily due to the encapsulation of doxorubicin within a liposomal vesicle, which evades detection by the mononuclear phagocyte system (MPS), thereby reducing the rate of clearance. Moreover, the PEGylation of the liposome surface further contributes to the ‘stealth’ properties of Doxil, allowing for enhanced circulation time and increased tumor exposure to the drug. This prolonged circulation leads to greater tumor drug accumulation due to the enhanced permeability and retention (EPR) effect, which is characteristic of many solid tumors, including those in multiple myeloma [60].

The unique properties of nanoparticles also allow for the simultaneous delivery of multiple therapeutic agents, opening up possibilities for effective combination therapies. A prime example is Vyxeos, a liposomal formulation that co-encapsulates cytarabine and daunorubicin in a fixed 5:1 molar ratio, allowing for optimized synergistic antileukemic effects [61].

Importantly, nanocarriers can reduce the toxicity of anticancer drugs by selectively targeting tumor cells and minimizing exposure to healthy tissues. Abraxane, an albumin-bound nanoparticle formulation of paclitaxel, has shown reduced toxicity compared to conventional paclitaxel, leading to improved patient tolerance [62].

Finally, by improving drug solubility and stability, nanoparticles can enable the formulation of oral and topical dosage forms of drugs that were previously only administrable intravenously, enhancing patient compliance. For example, Rapamune, an oral nanoparticle formulation of sirolimus, has greatly improved the ease of administration compared to intravenous forms [63].

In conclusion, nanotechnology-based treatments have demonstrated substantial benefits over traditional methods, and their continued development promises to revolutionize cancer treatment.

### 2.2. Nanoparticles for Cancer Imaging and Diagnosis

Nanotechnology has revolutionized cancer imaging and diagnosis, enhancing the capabilities of various imaging modalities and providing molecular-level information about tumors [64].

Nanoparticles can serve as powerful contrast agents due to their unique optical, magnetic, and radioactive properties. For instance, in magnetic resonance imaging (MRI), superparamagnetic iron oxide nanoparticles (SPIONs) have been used as contrast agents to improve signal intensity and spatial resolution, enabling the detailed visualization of tumors [65]. Similarly, in positron emission tomography (PET), radiolabeled nanoparticles can provide high-resolution images and quantitative information about tumor physiology [66].

Moreover, nanoparticles can be functionalized with targeting ligands to bind specifically to cancer biomarkers, allowing for the molecular imaging of tumors [67]. This targeted approach not only improves image contrast but also provides valuable information about the molecular characteristics of a tumor which can guide the choice of treatment.

Nanotechnology also holds promise for the early detection of cancer. For example, gold nanoparticles have been used in colorimetric assays for the detection of cancer biomarkers at extremely low concentrations, enabling the early diagnosis of cancer [68].

Additionally, the use of nanoparticles can facilitate the monitoring of treatment response. Changes in the size, morphology, or molecular characteristics of a tumor can be visualized using nanoparticle-enhanced imaging, providing real-time feedback on the efficacy of anticancer therapies [69].

In conclusion, nanotechnology has significantly advanced cancer imaging and diagnosis, offering the potential for early detection, precise molecular characterization, and effective monitoring of treatment response.

A detailed discussion of these topics will provide a solid foundation for understanding the potential and advantages of nanotechnology in cancer treatment, setting the stage for the subsequent sections that will delve into its application in multiple myeloma.

## 3. Review of Recent Advances in Nanotechnology for Myeloma

The application of nanotechnology in the field of myeloma treatment has generated considerable interest due to its potential to improve therapeutic outcomes. Nanotechnology, with its ability to manipulate materials at the nanoscale, opens up the possibility of creating finely tuned therapeutics that can target myeloma cells more specifically, increase the drug payload, and minimize off-target effects [70] (Figure 2).

Recent years have seen a surge in studies exploring nanotechnology-based treatments for myeloma. A notable example is the use of nanoparticle-based drug delivery systems, which have shown promise in improving drug bioavailability and reducing systemic toxicity [59]. For instance, liposomal nanocarriers have been used to encapsulate bortezomib, a proteasome inhibitor used in myeloma treatment, resulting in enhanced therapeutic efficacy in preclinical models [71]. Liposomal encapsulation of bortezomib has been shown to enhance its accumulation in the tumor microenvironment. This targeted delivery is achieved through the enhanced permeability and retention (EPR) effect, which is characteristic of many tumors. By exploiting this phenomenon, liposomal bortezomib demonstrates a higher therapeutic index compared to its free drug counterpart [72]. One of the major advantages of liposomal bortezomib is a reduction in systemic toxicity. The encapsulation prevents the widespread distribution of the drug, thereby decreasing the incidence and severity of side effects such as peripheral neuropathy, which is often seen with conventional bortezomib therapy [73]. The revised text further elaborates on how liposomal bortezomib can mitigate drug resistance mechanisms. For instance, the liposomal form is less susceptible to efflux by P-glycoprotein, one of the primary drug transporters implicated in the development of chemotherapy resistance [74].

Despite these advances, there is still a long way to go. The heterogeneity of myeloma and its complex interaction with the bone marrow microenvironment pose significant challenges in the development of effective nanotechnology-based therapies [75]. The complexity of intratumoral heterogeneity in multiple myeloma (MM) is a subject of considerable research interest. Genetic, epigenetic, and phenotypic variations within a single tumor can create a landscape of cancer cells with heterogeneous responses to therapy. This intratumoral diversity can result in the emergence of resistant subclones, thereby leading to treatment failure and disease progression [76]. As a result, there is a growing consensus among researchers and clinicians alike on the necessity to design combination treatment regimens that can simultaneously target multiple pathways and subclonal populations within MM [77]. The variability observed among MM patients, termed interpatient heterogeneity, presents a significant challenge for standardizing treatment protocols. The updated section delves into the implications of this heterogeneity for the management of MM. Personalized medicine has emerged as a crucial approach, allowing clinicians to devise treatment plans based on the unique genetic and phenotypic characteristics of each patient’s disease [78]. Identification of biomarkers through genomic and proteomic analyses has been pivotal in advancing this tailored approach, providing a framework for predicting response to treatment and disease outcomes [79]. Targeted therapy, designed to specifically attack cancer cells while sparing normal tissue, is complicated by the heterogeneity present in MM. Despite these challenges, advancements in molecular profiling have enabled the development of novel targeted therapies, such as liposomal bortezomib, that show promise in overcoming resistance mechanisms inherent in MM cell populations [74]. Research is increasingly focused on identifying synergistic combinations that can effectively target the molecular complexities of MM [80].

The nanotechnologies employed in myeloma treatment primarily revolve around different categories of nanoparticles and nanocarriers, each with unique properties that make them suitable for specific applications [45].

Liposomes, for instance, are spherical vesicles with an aqueous core surrounded by one or more phospholipid bilayers. Due to their biocompatibility and ability to encapsulate both hydrophilic and hydrophobic drugs, liposomes have been extensively used as nanocarriers in myeloma treatment. A prime example is liposomal doxorubicin, which has shown improved efficacy and safety compared to conventional doxorubicin [81]. In a Phase II clinical trial, liposomal doxorubicin demonstrated a 31% increase in overall response rate (ORR) compared to traditional formulations [80]. In addition, a multicenter study reported a safety profile for those receiving liposomal doxorubicin [82].

Polymeric nanoparticles offer another avenue for drug delivery. With their ability to protect drugs from degradation and deliver them in a sustained manner, polymeric nanoparticles have been used to deliver myeloma drugs like bortezomib [83]. The flexible design of these nanoparticles allows for the incorporation of targeting ligands, further enhancing their specificity to myeloma cells. One recent study focuses on advancing the treatment of multiple myeloma (MM) by overcoming the limitations of bortezomib (BTZ), a proteasome inhibitor hindered by chemoresistance and significant side effects. Researchers developed CD38-targeted chitosan nanoparticles (NPs) to improve the delivery and efficacy of BTZ. These NPs demonstrated a preferential release of BTZ within the tumor microenvironment, specific binding to MM cells, and increased cellular uptake of the drug, resulting in enhanced proteasome inhibition and cytotoxic effects against MM cells. The targeted NP system showed improved therapeutic efficacy and a lower toxicity profile in vivo, making it a promising approach for MM therapy with the potential to reduce side effects and circumvent resistance mechanisms [84]. In another study, researchers aimed to enhance the efficacy and specificity of bortezomib (BTZ) treatment in multiple myeloma (MM) by circumventing its common side effects and resistance issues. They developed nanoparticles encapsulating BTZ that were surface-functionalized with BCMA antibodies (BCMA-BTZ-NPs), ensuring targeted delivery to MM cells. The BCMA-BTZ-NPs showed improved uptake in BCMA-expressing cells, increased cytotoxicity, and overcame BTZ resistance by avoiding P-glycoprotein-related mechanisms. Furthermore, these nanoparticles were more effective in inducing apoptosis through mitochondrial depolarization, and they augmented immunogenic cell death and autophagic processes compared to the free drug. In vivo studies demonstrated tumor-specific accumulation, significant tumor reduction, and extended survival in a murine model. The findings indicate that BCMA-BTZ-NPs could significantly improve BTZ therapy for MM, offering a promising clinical advancement [85].

Dendrimers, highly branched and globular nanoparticles, have also found utility in myeloma treatment. Their well-defined structure and surface functionality allow for high drug-loading capacity and targeted delivery. One recent study examines the challenge of poor aqueous solubility of the anticancer drug bortezomib (BTZ) and proposes a solution using a dendrimeric platform, specifically PEGylated PAMAM dendrimers (BTZ-PEG-PAMAM), to enhance BTZ solubility and delivery. The BTZ-PEG-PAMAM dendrimers exhibited a particle size conducive to biological applications and high entrapment efficiency, and significantly increased the drug’s aqueous solubility (by over 68-fold). The in vitro release profile showed sustained drug release for up to 72 h. When tested against A549 and MCF-7 cancer cells, BTZ-PEG-PAMAM demonstrated superior efficacy with lower IC50 values compared to other formulations. This formulation also showed increased cellular uptake and, crucially, an 8.63-fold increase in bioavailability in Sprague Dawley rats compared to the pure drug. Overall, BTZ-PEG-PAMAM displays significantly improved pharmacokinetic parameters and holds potential as an effective delivery system for BTZ in cancer treatment [86]. In this study, researchers developed a novel BTZ prodrug-based nanoparticle using an amphiphilic PEGylated dendrimer with dopamine, aiming to overcome the limitations of bortezomib (BTZ) in treating solid tumors, such as poor stability and high toxicity. The dendrimer was synthesized by conjugating azide-functionalized polyethylene glycol with alkyne-functionalized dendrimer derived from 1,1-dimethylolpropionic acid and dopamine. These nanoparticles exhibited increased serum stability and released BTZ in acidic conditions, enhancing their effectiveness against subcutaneous tumors compared to BTZ alone. Furthermore, the targeting moiety c(RGDyK) was added to improve the nanoparticles’ specificity and anti-tumor efficacy, which showed promising results in both subcutaneous and intracranial tumor models. Notably, the nanoparticle formulation also reduced BTZ’s in vivo toxicity. These findings suggest that PEGylated BTZ dendrimer prodrug-based nanoparticles are a promising approach for solid tumor therapy [87]. Moreover, dendrimers have been used to deliver anti-myeloma drugs like melphalan, showing improved efficacy in preclinical studies [88].

Lastly, magnetic nanoparticles, often composed of iron oxide, serve dual roles in therapy and imaging. They can be guided to the tumor site using an external magnetic field and can be used for hyperthermia treatment or as contrast agents in MRI [65].

In summary, various types of nanoparticles and nanocarriers have been explored in the treatment of myeloma, each offering unique advantages in terms of drug delivery and specificity to the tumor site.

The efficacy of nanotechnology-based treatments for myeloma has been demonstrated in both preclinical and clinical studies, often showing potential improvements over conventional therapies [89].

Magnetic nanoparticles, when used in combination with external magnetic fields, have shown potential in targeted hyperthermia treatment. By heating the myeloma cells, these treatments can induce apoptosis and reduce tumor size [65].

Dendrimer-based drug delivery systems also represent a promising approach. For example, dendrimers carrying melphalan have shown improved drug delivery and enhanced therapeutic efficacy in preclinical myeloma models [88].

Taken together, these studies highlight the potential of nanotechnology-based treatments in improving myeloma outcomes. However, it is worth noting that while promising, these results are mostly based on preclinical studies, and more clinical trials are needed to validate these findings in patients.

**Figure 3:**
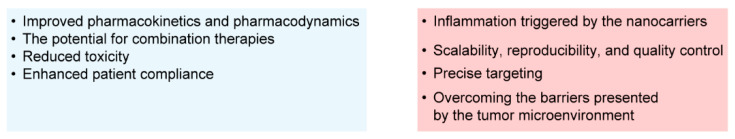
Potentials and side effects of nanotechnology.

The safety and efficiency of nanotechnology-based treatments for myeloma have been subjects of extensive study and are critical to their clinical application. In terms of safety, nanocarriers such as liposomes and polymeric nanoparticles have generally exhibited a favorable profile. They have shown reduced systemic toxicity due to their ability to encapsulate cytotoxic drugs and release them specifically at the tumor site (Figure 3).

Efficiency in nanotechnology-based treatments is characterized by factors such as drug-loading capacity, stability, and specificity of delivery. Polymeric nanoparticles and dendrimers often exhibit high drug-loading capacities, with their structure allowing for the encapsulation of a large amount of drug. Their design can also be tailored to optimize stability and sustained drug release, leading to enhanced therapeutic effect. For example, one study explores the development of high-loading-capacity polymeric nanoparticles (PNPs) for delivering bortezomib (BTZ), a proteasome inhibitor used in multiple myeloma treatment. Due to BTZ’s poor aqueous solubility and chemical instability, the research focuses on formulating N-(2-hydroxypropyl) methacrylamide (HPMA) copolymeric conjugates integrated with biotin for enhanced delivery and targeting. The synthesized HPMA-based conjugates, including HPMA-Biotin (HP-BT), HPMA-Polylactic acid (HPLA), and HPMA-PLA-Biotin (HPLA-BT), were thoroughly characterized and used to create PNPs with a narrow size distribution. These PNPs, particularly the biotinylated HPLA-BT variant loaded with BTZ, demonstrated significant anticancer activity against MCF-7 cells, with an IC50 value roughly half that of the pure drug, suggesting enhanced efficacy. The PNPs also had reduced hemolytic activity and showed increased cellular uptake attributed to the biotin tethering, which may improve selectivity and tumor targeting. In vivo pharmacokinetic studies revealed that the drug-loaded HPLA-BT PNPs had increased bioavailability and an extended half-life compared to BTZ alone. The findings indicate that the engineered HPMA-based PNPs offer a promising strategy for effective BTZ delivery in cancer therapy, with high drug-loading capacity and improved pharmacokinetics [90]. The use of targeting ligands can further improve the specificity of delivery to myeloma cells, minimizing off-target effects.

In summary, nanotechnology-based treatments for myeloma have demonstrated promising safety and efficiency profiles in preclinical and early clinical studies. However, further studies are required to fully understand and mitigate potential adverse events, and to optimize the efficiency of these treatments.

The preceding discussion underscores the considerable potential of nanotechnology-based treatments for myeloma, particularly in improving the safety and efficiency of drug delivery. These nanoparticle carriers, with their ability to encapsulate cytotoxic drugs and selectively deliver them to the tumor site, have demonstrated promising safety profiles and enhanced therapeutic efficacy.

However, challenges remain. While the safety profiles of these nanocarriers are promising, potential side effects, such as hypersensitivity and infusion-related reactions, require further investigation and mitigation [90]. Additionally, optimizing factors such as drug-loading capacity, stability, and specificity of delivery remain areas of ongoing research (Figure 3).

Looking forward, the landscape of nanotechnology-based treatments for myeloma is rapidly evolving. Ongoing research is exploring the use of more sophisticated nanocarriers, such as multifunctional nanoparticles and stimuli-responsive systems, which could further enhance the specificity and efficacy of drug delivery [91]. One recent study presents a drug delivery system using uniform and rigid polyhedral oligomeric silsesquioxane (POSS)–polymer conjugates, offering improved reproducibility over traditional polymeric vehicles. These nanoparticles (NPs) are multi-stimuli-responsive, reacting to ATP, acidic pH, and nucleophiles, and can encapsulate molecules like the fluorescent probe tetraphenylethylene (TPE) and the anticancer drug bortezomib (BTZ). The TPE@NPs enable visualization of uptake in tumor cells, while BTZ@NPs show selective cytotoxicity towards tumor cells. This platform could advance intelligent drug delivery systems for diagnostics and therapy due to its capacity to effectively deliver boronic acid-containing molecules with minimized systemic toxicity [92]. Furthermore, the integration of nanotechnology with emerging fields such as immuno-oncology and multi-omics could pave the way for personalized, precision therapies for myeloma [93]. As we continue to expand our understanding of myeloma at the molecular level, the future holds promise for more effective and tailored therapeutic approaches.

The integration of nanotechnology and immunotherapy represents a promising frontier in the treatment of myeloma. Immunotherapy, which involves harnessing the body’s immune system to fight cancer cells, offers a powerful and complementary strategy to traditional myeloma treatments [94]. However, the clinical efficacy of immunotherapy can be limited by challenges such as poor bioavailability, immunosuppressive tumor microenvironments, and systemic toxicity [95]. Herein lies the potential of nanotechnology. By encapsulating immunotherapeutic agents in nanocarriers, we can enhance their stability, improve their delivery to the tumor site, and help modulate the immune response [46]. In the following discussion, we will explore how nanotechnology can enhance immunotherapeutic strategies and review key studies in this exciting interdisciplinary field (Figure 2).

Nanotechnology holds considerable promise for enhancing immunotherapeutic strategies for myeloma. One major benefit of nanocarriers lies in their ability to protect immunotherapeutic agents from degradation and non-specific distribution, thus increasing their stability and bioavailability [51]. For instance, encapsulating immunotherapeutic agents within nanocarriers can protect them from premature degradation in the bloodstream, thereby prolonging their half-life and enhancing their therapeutic effect [96].

In addition, nanocarriers can be engineered to deliver immunotherapeutic agents specifically to the tumor site, reducing off-target effects and systemic toxicity [97]. This is crucial in myeloma, where tumor cells are often distributed within the bone marrow throughout the body. Nanocarriers can be designed to recognize and bind to specific molecular markers on myeloma cells, such as CD38, CD138, and BCMA, ensuring the targeted delivery of immunotherapeutic agents [91].

Furthermore, the controlled release of these agents from nanocarriers can be modulated, allowing for a sustained immune response over time. This can enhance the efficacy of immunotherapy and reduce the frequency of administration, improving patient compliance [98].

Several studies have highlighted the potential of utilizing nanotechnology in immunotherapy for myeloma. For instance, a preclinical study by Zhang et al. demonstrated that lipid-based nanoparticles encapsulating a PD-L1 antibody significantly inhibited myeloma tumor growth in a mouse model [99]. The nanoparticles were designed to release the antibody in response to the acidic tumor microenvironment, enhancing targeted delivery and reducing systemic toxicity.

In another study, Li et al. utilized a nanoparticle system to co-deliver a peptide vaccine and CpG adjuvant, an immunostimulant, to myeloma cells [100]. The study reported successful activation of dendritic cells and T-cells, leading to a robust anti-myeloma immune response. Notably, these findings suggest the potential of nanocarriers to not only deliver immunotherapeutic agents but also modulate the immune response.

In the evolving field of multiple myeloma (MM) treatment and diagnosis, recent innovations have showcased the integration of nanotechnology and bispecific antibodies to overcome the limitations of current clinical protocols.

A pivotal advancement in the diagnostic imaging of MM is seen with the development of a novel PET-based anti-BCMA nanoplatform labeled with ^64^Cu. This innovation trumps traditional imaging methods by enhancing the detection of small plasma cell populations within the bone marrow. The increased sensitivity and specificity provided by this platform could revolutionize the monitoring and management of MM, particularly in challenging areas such as the spine and femur, where accurate detection has historically been elusive [101].

On the therapeutic frontier, a notable leap forward is demonstrated through the creation of a CD138 receptor-targeting liposomal formulation that encapsulates the highly potent chemotherapeutic Mertansine (DM1). This approach effectively overcomes the severe toxicity issues associated with DM1, allowing for a higher dosage to be administered safely, significantly inhibiting tumor growth without noticeable systemic toxicity. This targeted delivery system represents a promising avenue to enhance the clinical applicability of potent anticancer drugs while mitigating adverse effects [102].

Turning to immunotherapy, the field is witnessing substantial progress with the introduction of B-cell maturation antigen (BCMA)-targeted peptide-encapsulated nanoparticles. These nanoparticles are adept at inducing a robust CTL response against MM, harnessing the potential of polyfunctional CTLs to improve anti-tumor efficacy. The sustained release and subsequent induction of memory CTLs highlight the potential of this strategy to provide durable therapeutic benefits [103].

Further enhancing the immunotherapeutic approach, the generation of an anti-PEG bispecific antibody (BsAb) that binds to PEG on liposomes and CD20 on lymphoma cells has been shown to significantly improve the delivery and efficacy of chemotherapeutic agents. This bispecific targeting leads to the creation of multivalent αCD20-armed liposomes which enhance cellular uptake and anticancer effects, presenting a strategic improvement in targeting hematologic malignancies [104].

Moreover, the advent of nanoparticle-based bispecific T-cell engagers (nanoBiTEs) and their multifaceted counterparts (nanoMuTEs) addresses the challenge of poor pharmacokinetics and single antigen targeting inherent in traditional CAR-T-cells and BiTEs. With an extended half-life facilitating less frequent dosing, these nano-engineered platforms demonstrate increased efficacy, particularly nanoMuTEs, which target multiple cancer antigens to preemptively thwart tumor escape via antigenic variation [105].

Clinical studies, while limited, have also shown encouraging results. A Phase I trial studying the safety and efficacy of nanoparticle-albumin-bound paclitaxel in combination with lenalidomide and dexamethasone in relapsed myeloma patients reported a favorable safety profile and a response rate of 78% [106].

However, while these studies are promising, they also underscore the challenges of translating preclinical findings to the clinic. Issues such as potential immunogenicity of nanocarriers, manufacturing scalability, and patient variability need to be addressed in future research.

The ability to modulate the immune system represents a key advantage of nanotechnology in the context of myeloma immunotherapy. Immune modulation involves adjusting the immune system’s response to improve its ability to recognize and destroy cancer cells. This can be achieved through a variety of mechanisms, including the suppression of immunosuppressive cells and the activation of effector immune cells [107].

Nanocarriers can be engineered to encapsulate and deliver agents that modulate the immune system, such as immune checkpoint inhibitors, cytokines, or immunostimulatory agents [108]. For instance, immune checkpoint inhibitors, which block proteins that prevent immune cells from attacking cancer cells, can be encapsulated within nanocarriers to enhance their delivery to the tumor site and reduce off-target effects [95].

By delivering these immune modulators in a targeted and controlled manner, nanocarriers can enhance the body’s immune response against myeloma cells. Importantly, this approach can also help overcome the immunosuppressive tumor microenvironment often observed in myeloma, which can inhibit the effectiveness of immunotherapies [109]. This precise control of the immune response afforded by nanotechnology holds significant promise for improving the efficacy and tolerability of immunotherapies for myeloma.

Despite the promise of nanotechnology in myeloma immunotherapy, several challenges remain. Potential side effects, such as inflammation triggered by the nanocarriers themselves, could limit their therapeutic window [45]. Moreover, manufacturing challenges, including scalability, reproducibility, and quality control of nanocarrier production, need to be addressed to ensure the successful clinical translation of these technologies [59].

Achieving precise targeting is another significant challenge. While nanocarriers can be engineered to recognize molecular markers on myeloma cells, such as CD38, CD1, BCMA, and CD138, tumor heterogeneity and the dynamic nature of these markers can complicate this strategy [110]. Additionally, overcoming the barriers presented by the tumor microenvironment, such as hypoxia and high interstitial pressure, requires innovative approaches [111].

Looking ahead, ongoing research is focused on optimizing nanocarrier design to enhance their targeting capabilities, stability, and biocompatibility. There is also increasing interest in integrating nanotechnology with other emerging fields in cancer therapy, such as gene therapy and the use of exosomes for drug delivery [112].

In conclusion, while challenges persist, the fusion of nanotechnology and immunotherapy offers a promising frontier in the quest for more effective myeloma treatments. By leveraging the unique properties of nanocarriers, we can improve the delivery and performance of immunotherapeutic agents, opening new avenues for the future of myeloma therapy.

## 4. Case Studies on Nanotechnology for Myeloma Treatment

As we navigate the complex landscape of myeloma treatment, real-world case studies provide invaluable insights into the practical applications of nanotechnology. In this section, we delve into specific instances where nanotechnology has been successfully integrated into myeloma therapy. The case studies discussed here offer a detailed examination of various nanomedicine-based therapeutic strategies, their clinical outcomes, and the challenges faced during their implementation. By dissecting these cases, we aim to extract key lessons that could inform future advancements in the field, bolstering our collective effort towards improved myeloma treatment.

For our initial case study, we turn our focus to the role of liposomal doxorubicin (Doxil) in the treatment of refractory myeloma. A landmark phase II clinical trial involving 50 patients set the stage [34]. In this study, patients with refractory myeloma were treated with a combination therapy of Doxil and bortezomib, a proteasome inhibitor. The results were promising: an overall response rate of 50% was observed, and the median progression-free survival extended to 6.5 months. However, this journey was not without obstacles. Managing side effects such as neutropenia and thrombocytopenia was a challenge, highlighting the need for careful patient monitoring and management during treatment. From this case, we glean important lessons on the potential of nanocarriers like liposomes to enhance the efficacy and safety profile of traditional chemotherapeutic agents, while also underscoring the need for vigilant management of associated side effects.

For the second case study, we explore the application of nanoparticle albumin-bound paclitaxel, commonly known as Abraxane, in the setting of relapsed myeloma. A phase I trial was conducted with 32 patients, where Abraxane was combined with lenalidomide and dexamethasone [106]. The results of the study were encouraging, with a response rate of 78% and a favorable safety profile. However, it was not all smooth sailing—the study highlighted potential challenges related to the use of nanoparticle-based therapies. For instance, the trial underscored the necessity of monitoring for side effects potentially associated with the nanocarriers themselves. This case study serves as an exemplar of how nanotechnology can enhance drug delivery and reduce off-target effects, thereby improving the therapeutic index of the treatment. Yet, it also underscores the need for careful monitoring and management of potential side effects associated with the nanocarriers.

In our third case study, we delve into the innovative use of polymeric micelles for targeted delivery of the proteasome inhibitor bortezomib in the treatment of myeloma. A pioneering preclinical study used poly(ethylene glycol)–polylactide (PEG–PLA) micelles to encapsulate bortezomib [113]. This nanoparticle formulation was found to enhance the cellular uptake and cytotoxicity of bortezomib in myeloma cells in vitro and showed improved anti-myeloma efficacy in vivo. Despite these promising results, the study pointed out potential challenges associated with stability and the controlled release of the drug from the micelles. This case study illustrates the significant potential of polymeric micelles in enhancing drug delivery to myeloma cells, but also emphasizes the need for further research to optimize the stability and controlled release characteristics of these nanocarriers.

The case studies showcased here underscore the transformative potential of nanotechnology in myeloma treatment. Liposomal doxorubicin and nanoparticle albumin-bound paclitaxel have demonstrated enhanced therapeutic profiles, while polymeric micelles have shown promise in augmenting the delivery of proteasome inhibitors. These case studies collectively contribute to our understanding of how nanocarriers can enhance drug delivery, improve safety profiles, and ultimately lead to better clinical outcomes.

However, these studies also bring to light the inherent challenges associated with nanocarrier-based therapies. These include the necessity of careful patient monitoring for potential side effects and the technical hurdles related to nanocarrier stability and controlled drug release. Future research efforts should thus be directed towards addressing these challenges. This could involve the exploration of novel nanocarrier designs, the development of strategies for effective side-effect management, and the investigation of approaches for ensuring stable and controlled drug release.

In conclusion, while the journey towards optimal nanomedicine-based myeloma therapies is fraught with challenges, the potential rewards are significant. As we continue to unravel the intricacies of nanotechnology, the horizon looks promising for its role in advancing myeloma therapy. The cases discussed herein not only underscore this potential but also provide a roadmap for leveraging nanotechnology to usher in a new era in myeloma treatment.

## 5. Future Perspectives and Potential Challenges

### 5.1. Novel Nanocarrier Design and Therapeutic Targets

**Figure 4:**
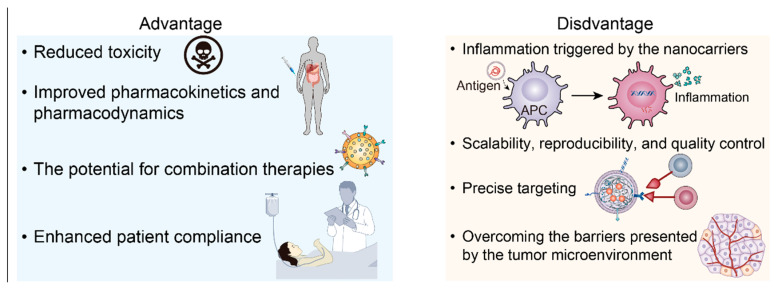
New era of nanotechnology.

As we look into the future of nanotechnology in myeloma treatment, several exciting prospects become apparent. A pivotal area of exploration lies in the design of innovative nanocarriers. Stimuli-responsive nanocarriers, capable of releasing their drug payload in response to specific cellular or tissue stimuli, hold immense promise [45]. This could potentially enhance the specificity of drug delivery and reduce off-target effects. Similarly, multifunctional nanocarriers equipped with both diagnostic and therapeutic capabilities could usher in a new era of theranostics in myeloma management (Figure 4).

Beyond nanocarrier design, there are opportunities for identifying novel therapeutic targets. As our understanding of myeloma biology deepens, new targets for nanomedicine intervention may emerge. This could pave the way for the development of targeted therapies that can disrupt the myeloma microenvironment or interfere with specific molecular pathways involved in myeloma pathogenesis.

Finally, the role of advanced technologies, such as artificial intelligence (AI), should not be overlooked. AI and machine learning algorithms can aid in the design of nanocarriers and the discovery of novel drugs for myeloma treatment [114]. By harnessing vast amounts of data, these technologies can expedite the process of drug discovery and optimize the design of nanocarriers for maximum therapeutic efficacy.

### 5.2. Integration of Multi-Omics

In the realm of precision medicine, leveraging multi-omics and spatial multi-omics is revolutionizing myeloma treatment by offering a detailed view across the genome, transcriptome, proteome, and metabolome. These approaches shed light on the intricate network of factors that drive myeloma, from genetic mutations to metabolic changes [115]. Adding spatial context, spatial multi-omics maps molecular characteristics to their physical locations within the tumor, illuminating the heterogeneity of myeloma and its microenvironmental interactions. This spatial insight is vital for crafting targeted therapies [116].

Nanotechnology, when combined with these omics approaches, holds great promise for personalized medicine. It can inform the creation of nanocarriers tailored to myeloma’s specific molecular landscape, enhancing treatment precision and effectiveness. Furthermore, novel therapeutic targets unveiled by these methods could lead to innovative nanomedicine strategies that target the myeloma microenvironment or disrupt key molecular pathways.

### 5.3. Targeting Aberrant Glycosylation in Multiple Myeloma

Aberrant glycosylation in multiple myeloma (MM), a complex post-translational modification frequently altered in cancer, is pivotal for disease progression and immune evasion. This modification involves changes in glycan structures, such as branching, sialylation, and fucosylation, often due to the overexpression of glycosyltransferases. These changes facilitate myeloma cell interactions with the bone marrow niche, modulate immune responses to promote immunosuppression, enhance cell adhesion properties, and ultimately support tumor growth and metastasis.

Therapeutic strategies to target these glycosylation anomalies include glycosyltransferase inhibitors, glycan-antagonists, lectin-based therapies, immunotherapies that target aberrant glycosylated cells, and enzymatic deglycosylation. Future research directions involve multi-omics and spatial multi-omics to understand glycosylation pathways, the development of glycan biomarkers for personalized medicine, and the investigation of resistance mechanisms to glycan-targeted therapies. The clinical translation of these strategies through rigorous trials could lead to novel, effective treatments for MM, highlighting the potential of targeting glycosylation aberrancies to improve patient outcomes [117].

### 5.4. Scientific, Clinical, and Manufactural Challenges

Despite the promising potential of nanomedicine-based therapies in myeloma treatment, numerous challenges persist in their clinical implementation. First and foremost, ensuring patient safety is paramount. While nanocarriers can improve the delivery of drugs, they may also introduce new safety concerns. For instance, the materials used in nanocarrier construction could elicit adverse biological responses, including cytotoxicity or inflammatory reactions [118]. Therefore, rigorous preclinical and clinical testing is essential to ensure the safety of these nanomaterials.

Another concern relates to the immunogenicity of nanocarriers. While the immune response to nanocarriers can be beneficial in some instances, for example, in the case of cancer vaccines, it can also lead to rapid clearance of the nanocarriers from the body or the induction of an undesired immune response [119]. Therefore, the design of nanocarriers must take into account their potential immunogenicity and develop strategies to minimize any adverse immune reactions.

Achieving effective drug delivery is hindered by formidable biological barriers, such as the blood–brain barrier and the cellular membrane. Overcoming these to ensure targeted delivery is complex and necessitates sophisticated nanocarrier designs informed by a thorough understanding of biological systems [45]. Scaling the production of nanocarriers from the lab to clinical settings presents substantial challenges. It requires not only the optimization of synthesis methods but also stringent quality control to ensure the scalability of these technologies [59]. Moreover, the heterogeneity of nanocarriers demands the standardization of their synthesis to ensure consistent quality and efficacy, a critical requirement for clinical trial validation and regulatory approval [46].

Speaking of regulatory approval, navigating the regulatory landscape is another significant hurdle. Regulatory bodies like the FDA and EMA have established guidelines for the development and approval of nanomedicine-based therapies, but these guidelines are continually evolving to keep pace with the rapid advances in nanotechnology [120]. Therefore, staying abreast of these changes and ensuring compliance is a critical part of the drug development process.

In conclusion, realizing the full potential of nanotechnology in myeloma treatment will require multidisciplinary collaboration. Scientists, clinicians, engineers, manufacturers, and regulatory bodies must all work together to overcome these hurdles and translate the promising potential of nanomedicine-based therapies into clinical reality.

## 6. Conclusions

This review has illuminated the transformative role of nanotechnology in advancing myeloma therapy. Through the detailed examination of advanced nanocarriers, we have uncovered their potential to fine-tune drug delivery, mitigate adverse effects, and enable precise treatment modalities. The synergy between nanotechnology and artificial intelligence stands out as a pivotal alliance, fostering the evolution of nanocarrier design and enhancing predictive models for patient treatment response.

Nanotechnology’s aptitude for refining drug delivery specificity stands as a revolutionary force in myeloma therapy. This precision not only curtails off-target consequences but also enhances treatment outcomes, marking a departure from generalized treatments to individualized care that could profoundly ameliorate patient prognoses. The horizon of nanotechnology’s application stretches into diagnostic realms, with the burgeoning field of theranostics marking a significant stride forward. Nanocarriers engineered to carry both therapeutic and diagnostic agents enable the concurrent monitoring of treatment and response, epitomizing the fusion of care and diagnostics.

In essence, nanotechnology harbors the capacity to redefine myeloma therapy through enhanced drug delivery precision, theranostic development, and the harmonization of omics-driven personalized treatment approaches. This innovative trajectory promises a future where therapy is not only more effective and safer but also uniquely tailored to each patient’s condition. With nanotechnology’s integration into multi-omics and spatial multi-omics, a new frontier in myeloma research emerges. These integrated approaches promise the discovery of novel targets and the genesis of personalized nanomedicine therapies, driving forward the quest for individualized care.

The future of myeloma treatment is thus not a distant dream but an evolving reality, with nanotechnology at its core. While challenges persist, the collective efforts of an interdisciplinary team spanning scientists, clinicians, engineers, and regulatory experts are paramount in translating these advancements from bench to bedside.
